# Using Ex Situ and In Situ HERFD-XANES to Reveal the Superior Oxidation and Reduction Cycling of Ceria Nanocubes Dispersed in Silica Aerogel

**DOI:** 10.1021/acs.jpcc.3c03785

**Published:** 2023-09-21

**Authors:** Lucy M. Morgan, Danilo Loche, Anna Corrias, Shusaku Hayama, Gavin Mountjoy

**Affiliations:** ‡ School of Chemistry and Forensic Science, University of Kent, Ingram Building, Canterbury CT2 7NH, U.K.; § Diamond Light Source, Harwell Science & Innovation Campus, Didcot OX11 DE, U.K.; ∥ School of Physics and Astronomy, University of Kent, Ingram Building, Canterbury CT2 7NH, U.K.

## Abstract

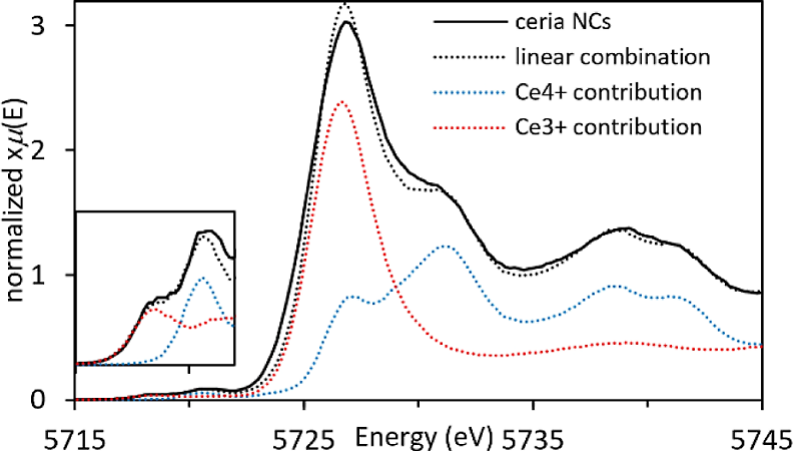

The oxygen storage capacity of ceria-based catalytic materials is influenced by their size, morphology, and surface structure, which can be tuned using surfactant-mediated synthesis. In particular, the cuboidal morphology exposes the most reactive surfaces; however, when the capping agent is removed, the nanocubes can agglomerate and limit the available reactive surface. Here, we study ceria nanocubes, lanthanum-doped ceria nanocubes, and ceria nanocubes embedded inside a highly porous silica aerogel by high-energy resolution fluorescence detection—X-ray absorption near edge spectroscopy at the Ce L_3_ edge. In situ measurements showed an increased reversibility of redox cycles in ceria nanocubes when embedded in the aerogel, demonstrating enhanced reactivity due to the retention of reactive surfaces. These aerogel nanocomposites show greater improvement in the redox capacity and increased thermal stability of this catalytic material compared to the surfactant-capped nanocubes. Ex situ measurements were also performed to study the effect of lanthanum doping on the cerium oxidation state in the nanocubes, indicating a higher proportion of Ce^4+^ compared to that of the undoped ceria nanocubes.

## 1 Introduction

The application of reducible oxides as catalysts is linked to the ability of the cations to change the oxidation states quickly and reversibly. Cerium dioxide (ceria) nanomaterials are well-established catalysts due to their exceptionally high reactivity, originating from the labile and reversible redox cycle between Ce^4+^ and Ce^3+^, as detailed in extended reviews.^1,2^ For example, ceria is widely used in CO_2_ conversion catalysis, including hydrogenation of CO_2_, activation of CO_2_ with alkanes, and nonreductive CO_2_ transformations.^3^ Ceria materials therefore have the capacity to store and release oxygen ions.

The redox obeys the equilibrium presented in eq 1 using the Kröger–Vink notation
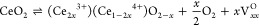
1This equilibrium shows the vacancy-driven mechanism by which ceria nanomaterials intake and release oxygen ions (oxygen storage capacity), depending on the partial pressure of oxygen in the environment. The oxygen storage capacity (OSC) is influenced by the morphology of the ceria nanoparticles and therefore can be enhanced by tuning the particle size and shape as well as by doping ceria.^4^ The OSC can be classified as total and dynamic OSC, with the former representing the thermal equilibrium amount of oxygen available and the latter the amount of oxygen kinetically available during fast transitions.

Ceria nanoparticles have been synthesized in cuboidal,^5^ spherical,^6^ polyhedral,^7^ and truncated octahedral^8^ shapes. The cuboidal ceria nanoparticles are mainly terminated by highly reactive ⟨100⟩ facets, giving them desirable morphologies for catalytic applications. Although highly reactive, this surface is not overly stable, so a capping agent is used in the synthesis process to stabilize the ⟨100⟩ surfaces and control the growth. The presence of the capping agent is undesirable for the catalytic activity of the nanocubes. As the capping agent used is usually organic and therefore thermally labile, it can be removed through thermal treatment. However, once the capping agent is removed, the nanocubes agglomerate, reducing the available ⟨100⟩ surface and thus lowering reactivity. Our group has successfully used silica aerogels to anchor ceria in its matrix (thus forming a nanocomposite),^9^ preventing agglomeration of the nanocubes. We have also been successful in effectively preserving the ⟨100⟩ surfaces of presynthesized nanocubes when they are embedded in a silica aerogel matrix, even after thermal treatment to remove the capping agent, as demonstrated by using powder X-ray diffraction and scanning transmission electron microscopy.^10^ N_2_ physisorption studies of the same samples also pointed out that these nanocomposites maintain the typical low density and high porosity of silica aerogels,^10^ which is likely responsible for the absence of strong interactions between the nanocubes and the silica. In fact, no evidence of ceria–silica crystalline phases was observed in these nanocubes embedded into silica aerogels even when thermally treated at 900 °C,^10^ in contrast to results obtained on ceria–silica samples prepared by coprecipitation.^11^

The reactivity of ceria nanoparticles can further be increased by incorporating dopants (Mn, Fe, Co, Ni, Cu, and Zn)^12^ into the lattice to form defects such as oxygen vacancies.^13,14^ Our previous studies demonstrated that the cubic fluorite structure of ceria is maintained with the incorporation of La^3+^ up to a doping concentration of 7.5 mol %, presenting remarkably high oxygen storage capacities.^13^

Several studies have employed X-ray absorption near edge spectroscopy (XANES) at the Ce L_3_ absorption edge to study the oxidation state and the redox properties of ceria-based materials also including in situ experiments.^13,15–21^ Here, we use high-energy resolution fluorescence detection (HERFD)-XANES at the Ce L_3_ absorption edge to investigate the oxidation state of Ce in synthesized ceria nanocubes, La-doped ceria nanocubes, and ceria–silica aerogel nanocomposites. HERFD-XANES has been shown to be much more sensitive^22–24^ to changes in the oxidation state than conventional XANES and valuable for the study of catalysts.^25,26^ In fact, HERFD-XANES has been used to evidence a reversible degree of reduction in cerium oxide ultrathin epitaxial films.^27^ Our recent paper^10^ presented HERFD-XANES spectra collected while oxidizing and reducing in situ at 275 °C ceria nanocubes and ceria nanocubes embedded in a silica aerogel that showed the effectiveness of silica aerogels in enabling thermally stable surfactant-free ceria nanocubes. In this work, HERFD-XANES measurements were carried out in a static environment at room temperature on ceria nanocubes, La-doped ceria nanocubes, and ceria–silica aerogel nanocomposites treated ex situ, and also during in situ oxidation/reduction cycles at 150, 275, and 400 °C on ceria nanocubes and ceria–silica aerogel nanocomposites. These results allow us to quantitatively determine the Ce-oxidation state and correlate this to the reactivity of the samples as a function of their composition and in situ environments.

## 2 Experimental Section

### 2.1 Experimental Synthesis of Nanocubes

The synthesis process followed is similar to that of Yang et al.^5^ and has also been used by Caddeo et al.^10^ and Loche et al.^13^ to obtain CeO_2_ and La-doped CeO_2_ nanocubes with the fluorite structure (2–5 nm in size^10^). All materials used were of analytical purity or higher. 15 mL portion of a 16.7 mmol L^–1^ water solution of Ce(NO_3_)_3_·6H_2_O (99.99%, Sigma-Aldrich) was placed in a Teflon-lined, stainless-steel autoclave (45 mL, Parr). To this solution were added 15 mL of toluene (HPLC grade, Fisher), 1.5 mL of oleic acid (extra pure, SLR, Fisher), and 0.15 mL of *tert*-butylamine (98%, Sigma-Aldrich). The autoclave was sealed (airtight) and transferred to a temperature-controlled electric oven, where it was subjected to a hydro-solvothermal treatment at 180 °C for 48 h before cooling to room temperature. The organic layer was then separated and purified by centrifugation to eliminate impurities. 30 mL of absolute ethanol (analytical grade, Fisher) was added to the purified solution to promote the precipitation of the nanocubes. The solid precipitate was removed and dried at room temperature in air overnight.

For lanthanum-doped nanocubes, La(NO_3_)_3_·6H_2_O (99.99%, Sigma-Aldrich) was mixed in proportional concentration with the Ce(NO_3_)_3_·6H_2_O (99.99%, Sigma-Aldrich) precursor as an aqueous solution in order to achieve 7.5 mol % La-doping. The same synthesis process as for the ceria nanocubes was then followed.

Catalysts need to be able to withstand relatively high working temperatures without structural degradation. Based on our previously published XRD and TEM work,^10^ the ceria nanocubes do retain their structure at increased temperatures when supported in a matrix but not when they are unsupported. To investigate whether the ceria nanocubes are affected by high working temperatures, unsupported ceria nanocubes and 7.5% La-doped ceria nanocubes were also thermally treated ex situ at 450 °C for 1 h prior to static HERFD-XANES measurements.

### 2.2 Synthesis of Ceria–Silica Aerogel Nanocomposites

The ceria–silica aerogel nanocomposites were prepared through a modified protocol previously used for the synthesis of silica-based aerogels.^28,29^ This consists of a 2-step acid–base-catalyzed sol–gel synthesis using the silicon alkoxide, tetraethoxysilane (TEOS, Si(OC_2_H_5_)_4_, 98%, Aldrich), as a precursor for the SiO_2_ phase. 7.9 mL of TEOS was added to a mixture containing 3 mL of absolute ethanol, and 3.965 mL acidic hydrolyzing solution (nitric acid (HNO_3_, 70%, Fisher Chemical) in absolute ethanol and distilled water). This was heated to 50 °C under reflux for 30 min to promote the hydrolysis of the TEOS. Upon cooling to room temperature, 1 mL of a dispersion of ceria nanocubes in toluene was then added in concentrations to obtain aerogel nanocomposites with 6 wt % of ceria nanocubes. A hydro-alcoholic solution of urea (NH_2_CONH_2_, >99.0%, Aldrich) in absolute ethanol and distilled water was then slowly added to the TEOS solution, stirring for 10 min before heating the solution to 85 °C under reflux until the solution viscosity changed. The sol was kept in an oven at 40 °C until gelation was complete. The gel was then submitted to high-temperature supercritical drying (up to 330 °C, 80 atm) in an autoclave (Parr, 300 cm^3^) filled with 70 mL of absolute ethanol.

The ceria wt % of the as-synthesized aerogel nanocomposites is determined by X-ray fluorescence (XRF, Panalytical Epsilon 3 Spectrometer), as reported in ref (10). This shows that the effective concentration of ceria nanocubes dispersed in the silica aerogel matrix is in good agreement with the target concentration.

The 6 wt % ceria–silica aerogel nanocomposite sample was also thermally treated ex situ at 450 °C for 1 h prior to static measurements, as done for the unsupported CeO_2_ and La-doped CeO_2_ nanocubes. In the case of the ceria–silica aerogel nanocomposite, the treatment at 450 °C also has the effect of removing organics (such as alkoxy groups) present on the surface of the aerogel after the supercritical drying process, as thermogravimetric analysis demonstrated in similar systems.^28,29^ A further ex situ treatment at 750 °C was carried out on the aerogel nanocomposite to determine if higher temperatures had any further effects. Since thermal treatment of unsupported nanoparticles at high temperatures is expected to produce extensive crystal growth,^10^ thermal treatment at 750 °C was not conducted on the unsupported CeO_2_ nanocubes and La-doped CeO_2_ nanocube samples.

Ce^4+^ and Ce^3+^ compounds (CeO_2_, 99.9%, Sigma-Aldrich and Ce(NO_3_)_3_·6H_2_O, 99.99%, Sigma-Aldrich, respectively), used as standards, were also analyzed to aid in determining the relative concentrations of Ce^3+^ and Ce^4+^ using a linear combination fit. (See Supporting Information for discussion of the choice of Ce(NO_3_)_3_·6H_2_O as a Ce^3+^ standard.)

It should be noted that the quantity of nanocubes synthesized in a single batch is limited. Therefore, several batches were combined into different samples to reach the required quantity for measurements.

### 2.3 HERFD-XANES Data Collection

High-energy resolution fluorescence detected (HERFD) X-ray absorption near edge spectroscopy (XANES) data at the Ce L_3_ absorption edge was collected using a 1 m diameter Rowland circle X-ray emission spectrometer at the Diamond Light Source on the I20-Scanning beamline.^30,31^ For these measurements, the spectrometer was equipped with three Si(4,0,0) spherical analyzer crystals and a Si drift detector, and all the HERFD spectra were taken with the spectrometer tuned to the maximum of the Ce Lα_1_ fluorescence line, which corresponds to an angle of 70.66°.^32^ Ce L_3_ HERFD-XANES is probing the excitation of the 2p_3/2_ core electrons to the unoccupied 4f and 5d states. Data analysis was performed using the ATHENA software,^33^ with the relative concentrations of Ce^3+^ and Ce^4+^ determined using the linear combination fitting tool in ATHENA^33^ and the HERFD-XANES spectra of the Ce^3+^ and Ce^4+^ standards. Kvashnina et al. measured the HERFD-XANES spectrum of CeO_2_ with Ge(331) analyzer crystals, which provide a better energy resolution than our experiment because of the higher spectrometer angle (87°).^34^ Nevertheless, with the resolution in our HERFD-XANES experiment, the spectra of the standards are still showing much greater details than those of the spectra of the standards obtained by conventional XANES^13^ since the core-hole lifetime of the Lα_1_ fluorescence line is relatively large (∼3.4 eV). In fact, in the conventional XANES spectra, pre-edge features are barely visible, and post-edge features are much broader.^13^

Samples were made into pellets for static room temperature measurements. Pellets were prepared by homogenizing 100 mg of polyvinylpyrrolidone (PVP) with the sample using an agate mortar and pestle. Ce^3+^ and Ce^4+^ standards were added to PVP as powders (4 mg), while a dispersion of nanocubes in toluene was added to the PVP and toluene left to evaporate off, and afterward, a homogeneous powder was obtained. Once homogenized, the pellet mixture was compacted into a pellet using a press. Static room temperature measurements were performed using the pellet samples placed in a sample stage where the flat surface was 45° to the incident beam and spectrometer. (See Supporting Information for discussion of self-absorption being a minor effect.)

For in situ measurements at variable temperatures, samples were loaded into a Kapton capillary. Capillaries were prepared by filling the center of a Kapton tube (Durafilm, 1.62 mm outer diameter, 1.46 mm internal diameter, and 80 μm wall thickness) with ∼1 cm length of sample. The aerogel nanocomposite and CeO_2_ standards were used as powders without the need for additional manipulation. The nanocubes were impregnated into quartz wool from a dispersion of the nanocubes in toluene. Each sample was bracketed at each end with quartz wool to hold it in place. In situ measurements were made during oxidation (5% O_2_ in He) and reduction (5% H_2_ in He) cycles at room temperature, 150, 275, and 400 °C using a plug-flow reactor cell with Kapton tubes. The Kapton tube was flushed with N_2_ for 10 min initially and between measurements due to the potential ignition of H_2_/O_2_ mixtures at high temperatures. Each gas flow rate was 20 SCCM (standard cubic centimeters per minute), and the temperature was increased by roughly 1 °C/s. A reduction in temperature, back to room temperature, was achieved by removing the heat source, which took longer to cool (>1 h). Measurements were taken within a few minutes of reaching the desired temperature under the desired flow of gas (O_2_/H_2_).

As there is some natural variation in the Ce^3+^/Ce^4+^ ratio arising from the synthesis process, the initial HERFD-XANES measurements of the samples may differ slightly. External conditions, such as time under the beam and under N_2_, have also been shown to affect this ratio. However, the Ce^3+^/Ce^4+^ ratios become equivalent after initial oxidation at 150 °C, which provides enough thermal energy and sufficient time for atmospheric effects to stabilize the Ce^3+^/Ce^4+^ ratios. The effect of time under the beam may still be present but appears less prominent. (See Supporting Information for a discussion of reducing beam damage effects.).

## 3 Results

### 3.1 Static Room Temperature Measurements

**Figure 1:**
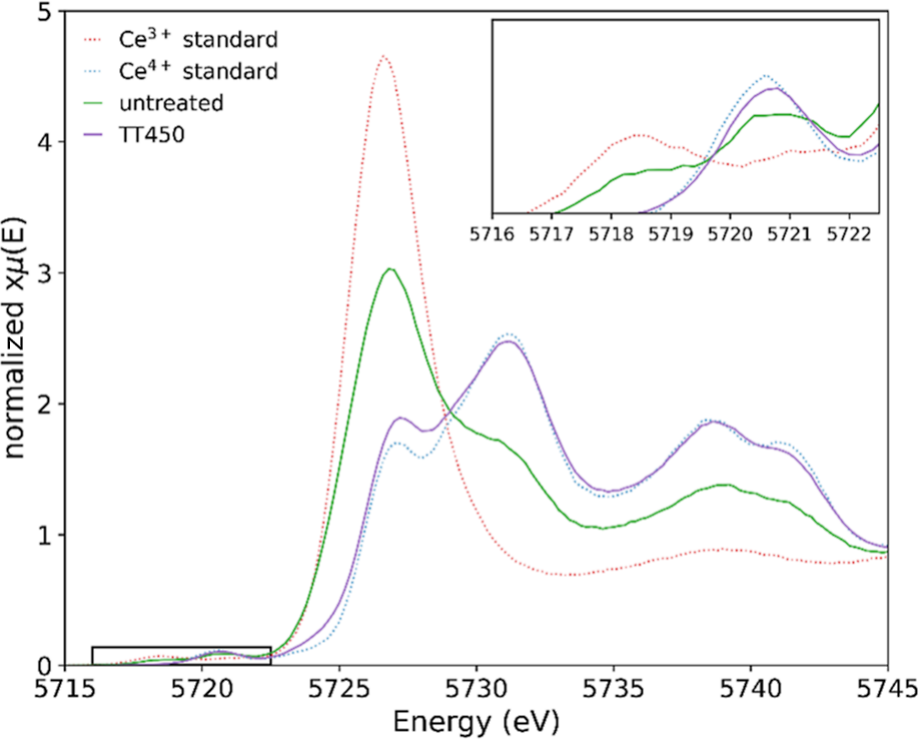
HERFD-XANES Ce L_3_-edge spectra of ceria nanocubes at room temperature (green) and thermally treated ex situ at 450 °C (purple), along with the Ce^3+^ (dotted red) and Ce^4+^ (dotted blue) standards. The inset presents a magnified pre-edge region.

Ceria nanocubes have the same fluorite structure (fcc) as that of pure microcrystalline Ce(IV) oxide. The latter has an ideal composition comprising solely a Ce^4+^ oxidation state. However, microstructural features formed during the synthesis of nanocubes create oxygen vacancies, which lead to a mixture of Ce^3+^ and Ce^4+^. The HERFD-XANES Ce L_3_-edge spectra of the synthesized ceria nanocubes and the Ce^3+^ and Ce^4+^ standards, at room temperature, are presented in Figure 1. For the Ce^3+^ (red) and Ce^4+^ (blue) standards, it is noted that there is a shift in edge position correlated with the oxidation state. Moreover, changes in peak intensities provide valuable information about the relative amounts of Ce^3+^ and Ce^4+^. In fact, there is a significant reduction in peak intensity at 5727 eV for Ce^4+^ which also presents an additional peak at 5732 eV (both of these peaks are due to 2p to 5d transitions).^35^ The ratio between the 5727 and 5732 eV peaks can therefore be used to determine the percentage of Ce^3+^ and Ce^4+^ in the samples. A shift to higher energy in the pre-edge peak (∼5720 eV, due to 2p to 4f transitions) is also observed, presenting an additional indication of a change in oxidation state.^35^ For the ceria nanocubes (green), it is noted from the presence of peaks at 5727 and 5732 eV that both Ce^3+^ and Ce^4+^ are present, which is further evidenced in the magnified pre-edge region. The relative concentrations of Ce^3+^ and Ce^4+^ in the ceria nanocubes were calculated using the linear combination fitting tool in ATHENA. This was performed using Ce(NO_3_)_3_·6H_2_O and CeO_2_ as standards for Ce^3+^ and Ce^4+^, respectively, obtaining relative concentrations of 48% Ce^3+^ and 52% Ce^4+^. The fitting results are shown in the Supporting Information (Figure S1).

Under ambient conditions, the ceria nanocubes are capped by a surfactant (oleic acid), which is used in the synthesis to control the growth of the nanoparticle surfaces. This allows the formation of the metastable cubic morphology with exposed {100} reactive surfaces and prevents nanoparticle aggregation. The surfactant, however, also acts as a barrier, which can inhibit surface activity. Removal of the surfactant by thermal treatment should therefore allow for improved surface activity, and as oleic acid is an organic material, it is thermally labile. The same thermal treatment can, however, produce the aggregation of nanocrystals and their growth. Thermal treatment ex situ at 450 °C is expected to be sufficient to cause decomposition and remove the surfactant from the nanocubes. Figure 1 also presents the HERFD-XANES Ce L_3_-edge spectra of the ceria nanocubes after thermal treatment ex situ at 450 °C (purple). The difference between the as-synthesized and thermally treated ceria nanocubes is very evident from visual inspection, where the thermally treated ceria nanocubes show almost the same results as the Ce^4+^ standard. The relative concentrations of Ce^3+^ and Ce^4+^ in the thermally treated ceria nanocubes are 4 and 96%, respectively, in comparison to 48 and 52% in the as-synthesized nanocubes. It should be pointed out that previous findings from our group^10^ showed that the nanocubes aggregate and grow when thermally treated at 450 °C as a consequence of the capping agent being degraded.

**Figure 2:**
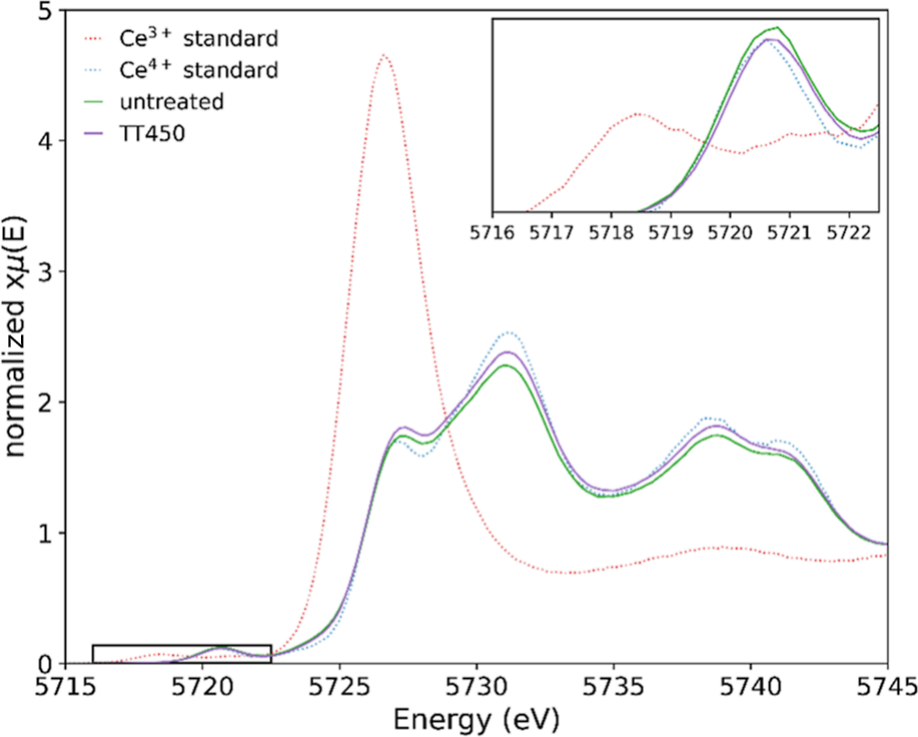
HERFD-XANES Ce L_3_-edge spectra of 7.5% La-doped ceria nanocubes as-synthesized (green), thermally treated ex situ at 450 °C (purple), and the Ce^3+^ (dotted red) and Ce^4+^ (dotted blue) standards. The inset presents a magnified pre-edge region.

Doping the ceria nanocubes with La^3+^ promotes the formation of oxygen vacancies^13^ because of the differences in the oxidation states between La^3+^ and Ce^4+^. Figure 2 presents the HERFD-XANES Ce L_3_-edge spectra of the 7.5% La-doped ceria nanocubes as-synthesized (green) and after thermal treatment ex situ at 450 °C (purple) along with the Ce^3+^ (red) and Ce^4+^ (blue) standards. Here, the cerium in the La-doped nanocubes is mostly Ce^4+^, with the relative concentrations of Ce^3+^ and Ce^4+^ for the as-synthesized nanocubes measured at 5 and 95%, respectively. This presents a higher relative concentration of Ce^4+^ compared to the undoped ceria nanocubes (49%), and similar relative concentrations to those of the thermally treated undoped ceria nanocubes (96%). Thermal treatment of the 7.5% La-doped ceria nanocubes (Figure 2, purple) presents similar relative concentrations to those of the as-synthesized equivalent, suggesting removal of the surfactant had no further effect on the Ce-oxidation state. The remaining 4–5% Ce^3+^ may be unable to be oxidized in these conditions, requiring higher temperatures and/or higher dopant concentrations to fully oxidize.

**Figure 3:**
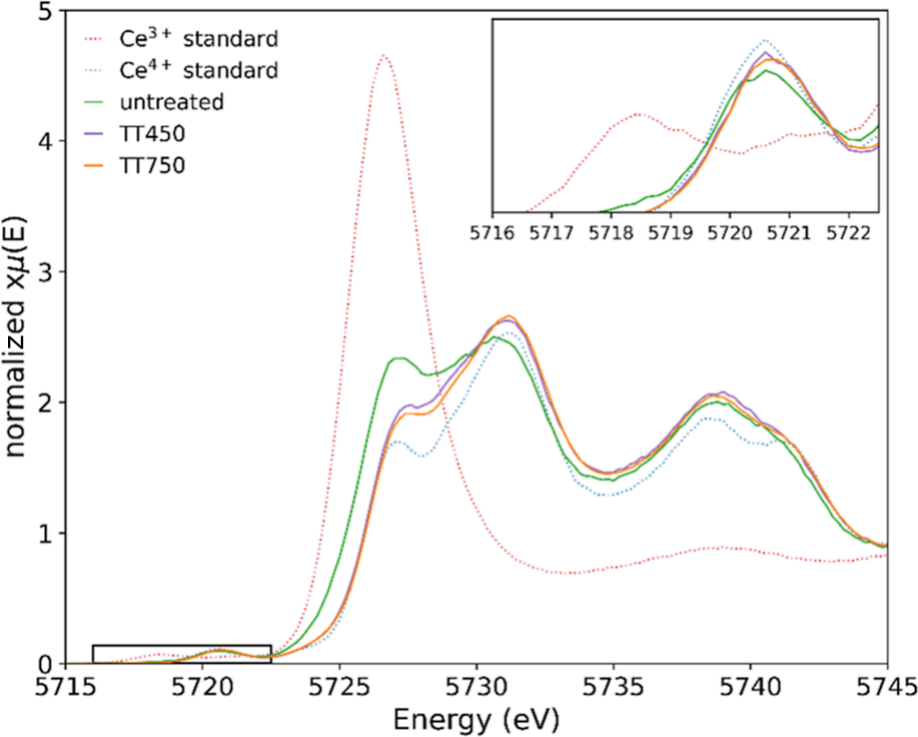
HERFD-XANES Ce L_3_-edge spectra of 6 wt % ceria–silica aerogel nanocomposite as-synthesized (green), thermally treated ex situ at 450 °C (purple), thermally treated ex situ at 750 °C (orange), and the Ce^3+^ (dotted red) and Ce^4+^ (dotted blue) standards. The inset presents a magnified pre-edge region.

Placing the ceria nanocubes inside a support matrix, i.e., silica aerogel, should prevent aggregation of the nanoparticles and allow for retention of the cuboid shape, even when thermally treated at a high temperature. The HERFD-XANES Ce L_3_-edge spectra of 6 wt % ceria–silica aerogel nanocomposites, as-synthesized (green), after thermal treatment ex situ at 450 °C (purple), and after thermal treatment ex situ at 750 °C (orange), are presented in Figure 3. The relative concentrations of Ce^3+^ and Ce^4+^ for the as-synthesized aerogel nanocomposite are 14 and 86%, respectively, and for the thermally treated aerogel nanocomposites (at both 450 and 750 °C), 0–1% and 99–100%, respectively. Dispersion of the ceria nanocubes in a support matrix increased the Ce^4+^ relative concentration from 52% (unsupported) to 86%. Further to this, when thermally treated ex situ, almost all Ce ions were Ce^4+^.

The capacity of metal oxides, such as ceria, for oxidation and reduction is dependent on variables such as the chemical composition and supporting substrates. Understanding these specific variables is, therefore, vital to furthering the capacity of oxides as catalysts. From these static measurements, we have shown that doping ceria nanocubes with lanthanum increases the relative concentration of fully oxidized Ce^4+^, in comparison to the undoped nanocubes. Similarly, the use of silica aerogel as a support matrix aids in preventing aggregation of the ceria nanocubes, which also increases the relative concentration of fully oxidized Ce^4+^. Using thermal treatment to remove the surfactant from the nanocube surfaces is highly effective at promoting oxidation to Ce^4+^, with the combination of thermal treatment and supporting the ceria nanocubes in an aerogel nanocomposite providing almost complete Ce-oxidation to Ce^4+^. A summary of the measured samples and their relative concentrations of Ce^3+^ and Ce^4+^ are presented in Table 1.

**Table 1:** Estimated Relative Concentrations of Ce^3+^ and Ce^4+^ within the Ceria Nanocubes, 7.5% La-Doped NCs, and the 6 wt % NCs in Aerogel Nanocomposite, As-Synthesized and after Ex Situ Thermal Treatment during Static Room Temperature Measurements

		concentration (%)	
	thermal treatment	Ce^3+^	Ce^4+^
NCs	none	48	52
	450°C	4	96
7.5% La-doped NCs	none	5	95
	450°C	4	96
6 wt % NCs in aerogel	none	14	86
	450/750 °C	≤1	≥99

### 3.2 In Situ Measurements

In situ measurements of the undoped ceria nanocubes and ceria–silica aerogel nanocomposites were conducted to investigate the redox properties of the material under operational working conditions. These measurements investigated the effects of the reduction/oxidation cycle at increasing temperatures and the reversibility of the oxidation step.

The in situ environment was first used on the Ce^4+^ standard to determine any specific effects of the gas environment on a sample where minimal to no changes would be expected. Measurements started at room temperature under N_2_ gas (neutral) and then heated at a rate of 1 °C/s to 400 °C under H_2_ gas (reduction), followed by oxidation with O_2_ before reducing the temperature back to ambient under N_2_. It was noted at this point that initial measurements under N_2_ resulted in an increase in Ce^3+^, suggesting energetically removable oxygen is present on the surface under a gas flow. The percentage of Ce^3+^ continued to increase with extended time under N_2_; however, the limits of this were not investigated within this study.

For the Ce^4+^ standard, at each stage, there was a small change in the determined relative ratios of Ce^3+^ and Ce^4+^, ranging between 91 and 95% Ce^4+^. This change was attributed to the constant gas flow detaching some of the more easily removable oxygen from the surface. The HERFD-XANES Ce L_3_-edge spectra of the in situ Ce^4+^ standard and a table with relative ratios of Ce^3+^/Ce^4+^ can be found in the Supporting Information (Figure S2 and Table S1).

A first in situ experiment on ceria nanocubes was carried out by collecting the HERFD-XANES Ce L_3_-edge spectra at room temperature, 150, 275, and 400 °C, under both reduction and oxidation conditions, which are given in Supporting Information (Figures S3 and S4 and Table S2). The temperatures, conditions, and relative ratio of Ce^3+^/Ce^4+^ are presented in Table 2. It should also be noted that this sample was held under N_2_ gas flow for an extended period, in comparison to the other samples, due to additional setup testing; therefore, the initial quite large Ce^3+^/Ce^4+^ ratio might be influenced by a combination of different factors such as the extended gas flow, extended time under the beam (i.e., beam damage),^36^ natural variations in the synthesis process, and the sampling location. There is a clear indication of reversible reduction at 150 °C, with Ce^4+^ concentrations decreasing from 52 to 28% under reducing conditions and then increasing to 44% after oxidizing conditions. The nanocubes were then re-reduced at 275 °C to 31% Ce^4+^ concentration and reoxidized to 96%. The considerably higher oxidation is likely due to a decomposition of the thermally labile surfactant, thus removing it from the nanocube surface, which is corroborated by the TGA data previously given in Loche et al.^13^ The oxidation stage at 275 °C was also much slower to stabilize than at 150 °C, taking around 1 h 40 min rather than a few minutes, as shown in Figure S5, supporting the interpretation of the surfactant being slowly and progressively removed at 275 °C under oxidizing conditions.

**Table 2:** Estimated Relative Concentrations of Ce^3+^ and Ce^4+^ within the Ceria Nanocubes during In Situ Measurements Taken at Increasing Temperatures and under Alternating Reduction/Oxidation Conditions

		concentration (%)	
ceria nanocubes	gas flow	Ce^3+^	Ce^4+^
RT	N_2_	48	52
150°C	H_2_	72	28
150°C	O_2_	56	44
275°C	H_2_	69	31
275°C	O_2_	4	96
400°C	H_2_	33	67
400°C	O_2_	3	97
RT	N_2_	1	99

With the surfactant removed, the nanocubes are more readily able to oxidize, which results in a higher percentage of Ce^4+^. Without the surfactant supporting the nanocuboidal shape, there may be a change in morphology and/or particle aggregation, as has previously been reported for similar unsupported ceria nanocubes.^10^ Attempts of reduction at a higher temperature (400 °C) resulted in 67% Ce^4+^ concentration, showing some reduction but not as significant as the lower temperatures, which further supports a likely change in particle morphology and/or aggregation, i.e., less reactive surfaces. When reoxidized, the ceria nanoparticles returned to 97–99% Ce^4+^.

The removal of the surfactant at 275 °C leaves the surfaces fully exposed and active to oxidation while also potentially negatively affecting the morphology and reducibility of the nanoparticle. Without the protection of the surfactant, oxidation is the more energetically favorable process, and activating reduction after oxidation is likely to require additional energy. A second in situ experiment on a fresh sample of nanocubes was therefore carried out to study in more detail the reversibility of the redox process once the surfactant is removed. HERFD-XANES Ce L_3_-edge spectra of the fresh ceria nanocubes were collected, starting at room temperature and then increasing directly to 275 °C, followed by 400 °C. At each temperature, the nanoparticles were reduced, oxidized, and reduced a second time. As previously mentioned, the Ce^3+^/Ce^4+^ ratio at room temperature is influenced by the time under the beam/N_2_ gas flow, synthesis differences, and sampling location. Due to less time under the beam/N_2_ gas flow, the sample is less reduced compared to the first in situ measurement at room temperature. The oxidation of the nanocubes at 275 °C is again gradual, taking around 1 h to stabilize, with 93% Ce^4+^, similar to the previous equivalent measurement (i.e., Table 2, 275 °C shows 96% Ce^4+^). After this oxidation step, the Ce^3+^/Ce^4+^ ratio realigns with the ratios of the first in situ sample.

**Figure 4:**
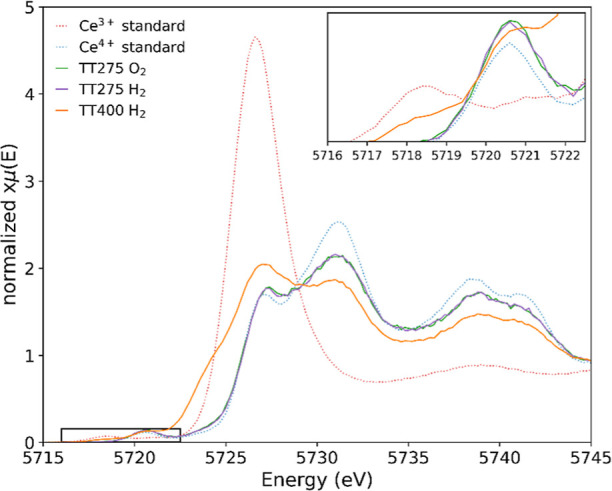
HERFD-XANES Ce L_3_-edge spectra of ceria nanocubes under in situ oxidation at 275 °C (green), followed by reduction at 275 °C (purple), then reduction at 400 °C (orange), and the Ce^3+^ (dotted red) and Ce^4+^ (dotted blue) standards. The inset presents a magnified pre-edge.

Figure 4 presents the HERFD-XANES Ce L_3_-edge spectra of the ceria nanocubes after oxidation at 275 °C (green) and after the second reduction at 275 °C (purple), followed by reduction at 400 °C (orange). Here, it is clearly shown that a repeated attempt to reduce the nanocubes while keeping the temperature constant after the oxidation step was unsuccessful, with reduction only occurring with an increase in temperature, i.e., additional thermal energy. The nanocubes were then oxidized at 400 °C, resulting in 94% Ce^4+^, and then re-reduced. This resulted in a reduction without the need for additional thermal energy; however, it was unable to achieve the same results as the first reduction, e.g., only 12% Ce^3+^ compared to 29% Ce^3+^. A summary of the percentage oxidation states for nanocubes is presented in Table 3.

**Table 3:** Estimated Relative Concentrations of Ce^3+^ and Ce^4+^ within the Ceria Nanocubes during the Second In Situ Measurements Taken at Increasing Temperatures and under Reduction/Oxidation Conditions

		concentration (%)	
ceria nanocubes	gas flow	Ce^3+^	Ce^4+^
RT	N_2_	26	74
275°C	H_2_	40	60
275°C	O_2_	7	93
275°C	H_2_	7	93
400°C	H_2_	29	71
400°C	O_2_	6	94
400°C	H_2_	12	88

Ceria–silica aerogel nanocomposites should prevent aggregation of the nanoparticles and allow for the retention of the cuboid shape after removal of the surfactant. In situ measurements of a 6 wt % ceria–silica aerogel nanocomposite were conducted at 150, 275, and 400 °C, both under reduction and oxidation conditions. A second reduction step was conducted at 275 °C before raising the temperature and again at 400 °C to investigate the re-reduction properties at higher temperatures, similarly to the second measurement on the nanocubes.

The initial in situ room temperature measurement of the aerogel nanocomposite gave a Ce^4+^ concentration of 76% Ce^4+^ concentration. When heated to 150 °C under H_2_, the nanocubes were reduced to 63% Ce^4+^, showing a change similar to that of the unsupported nanocubes. Oxidation at this temperature resulted in 81% Ce^4+^, higher than that at room temperature. The aerogel nanocomposite was then reduced at 275 °C, resulting in 62% Ce^4+^, similar to that measured at 150 °C. Again, the following oxidation at 275 °C was gradual, likely due to the removal of the surfactant; however, there are clear differences in the time frame of this process compared to the unsupported nanocubes, i.e., the most notable oxidation occurred in 30 min compared to 1 h 30 min for the unsupported nanocubes. This shorter time frame could be due to the separation of the nanocubes, making removal of the surfactant easier, and/or related to the surfactant potentially being partially removed during the aerogel synthesis. However, the slow oxidation is still attributed to the progressive removal of the surfactant from the nanocube surfaces. The HERFD-XANES Ce L_3_-edge spectra of these measurements are given in Supporting Information (Figure S6). Once stabilized, the oxidation of the aerogel nanocomposite resulted in 93% Ce^4+^.

**Figure 5:**
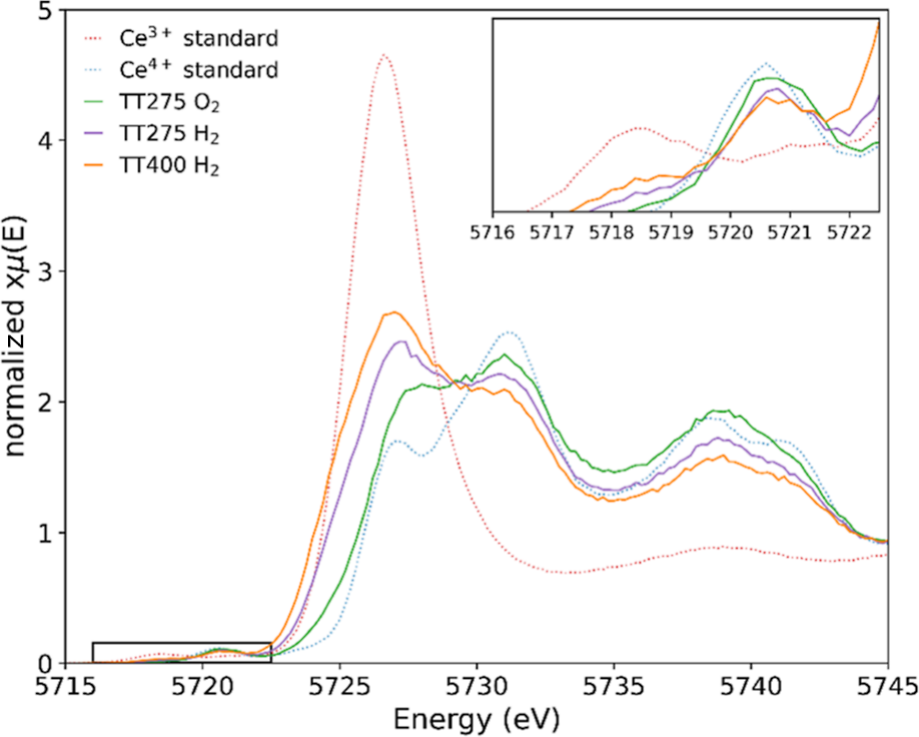
HERFD-XANES Ce L_3_-edge spectra of the ceria–silica aerogel nanocomposite under in situ oxidation at 275 °C (green), followed by reduction at 275 °C (purple), then reduction at 400 °C (orange), and the Ce^3+^ (dotted red) and Ce^4+^ (dotted blue) standards. The inset presents a magnified pre-edge.

The reversibility of the redox cycle of the ceria–silica aerogel nanocomposite at 275 °C was investigated by performing a second reduction step in situ at 275 °C before additional thermal energy was introduced. We observed that the Ce^3+^ concentration increased from 7 to 27%, showing that the oxidation is, at least partly, reversible at 275 °C without the requirement of additional thermal energy. This suggests the dispersion of nanocubes within a host matrix has prevented particle aggregation and aided in stabilizing the nanoparticles reduction/oxidation activity at higher thermal working conditions after the removal of the surfactant. With the addition of thermal energy (400 °C), the reduction is improved, further increasing the Ce^3+^ concentration to 39%. The HERFD-XANES Ce L_3_-edge spectra showing the re-reduction of cerium in the aerogel nanocomposite at 275 °C and further reduction at 400 °C are given in Figure 5.

**Figure 6:**
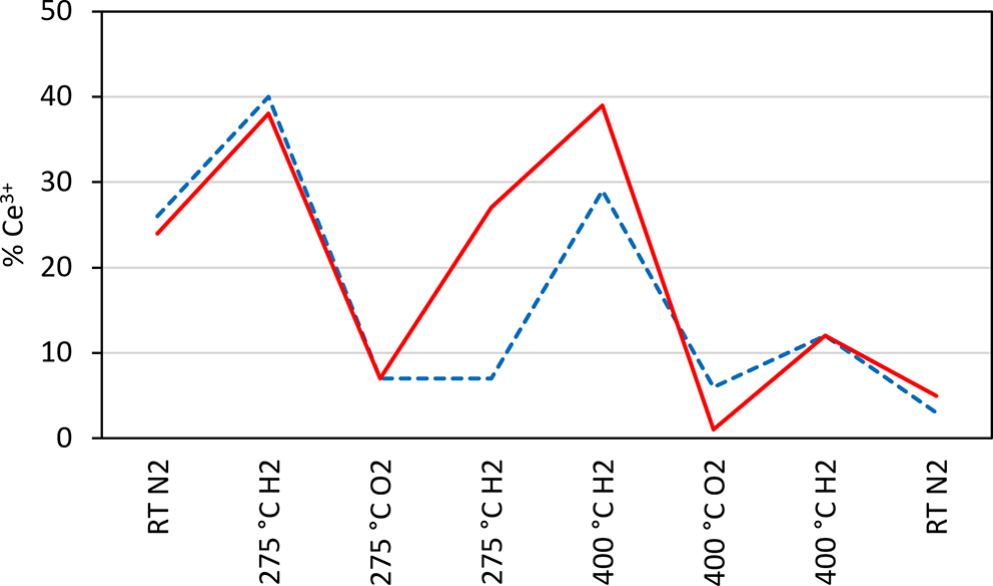
Estimated relative concentrations of Ce^3+^ for unsupported CeO_2_ nanocubes (dashed line) and ceria–silica aerogel nanocomposites (solid line) during in situ measurements taken at increasing temperatures and under alternating reduction/oxidation conditions (see Tables S1, 3, and 4).

The aerogel nanocomposite was then oxidized and reduced at 400 °C, giving almost complete oxidation and a similar reduction to the unsupported nanocubes. Table 4 contains a summary of the percentage of oxidation states for Ce within the ceria–silica aerogel nanocomposite. Figure 6 provides a comparison of the in situ results for the unsupported nanocubes (Table 3) and ceria–silica nanocomposite (Table 4). Here, the notable increase in oxidation at 275 °C, attributed to the removal of the surfactant, can be observed, along with the improved re-reducibility of the nanocubes dispersed in aerogel in comparison to the unsupported nanocubes. However, after an oxidation cycle at 400 °C, the concentration of Ce^3+^ produced (12%) is the same for both materials, suggesting that the advantages of the nanocomposite may be limited in applications at higher temperatures.

**Table 4:** Estimated Relative Concentrations of Ce^3+^ and Ce^4+^ within the 6 wt % Ceria–Silica Aerogel Nanocomposites during In Situ Measurements Taken at Increasing Temperatures and under Alternating Reduction/Oxidation Conditions

		concentration (%)	
nanocomposites	gas flow	Ce^3+^	Ce^4+^
RT	N_2_	24	76
150°C	H_2_	37	63
150°C	O_2_	19	81
275°C	H_2_	38	62
275°C	O_2_	7	93
275°C	H_2_	27	73
400°C	H_2_	39	61
400°C	O_2_	1	99
400°C	H_2_	12	88

## 4 Discussion

Redox reactions in catalysts require the formation of oxygen vacancies, a crucial parameter of which is the energy of the vacancy. The vacancy formation energy needs to be as low as possible but also large enough to be energetically favorable to create and refill vacancies. This ability is linked with the electronic structure of the material, which has drawn great interest.^4,37,38^ Through the analysis of the cerium oxidation states under redox conditions, we were able to gain insight into the evolution of oxygen vacancies and the effects caused by temperature and surfactant removal.

Catalytic working conditions include thermal energy; therefore, the ability to remain active at higher temperatures is crucial to its performance. Cuboidal ceria particles have the most reactive morphology due to their highly reactive {100} surface. This morphology is possible only through the use of a surfactant, which also prevents aggregation of the nanoparticles that would otherwise reduce the active surface area. As the surfactant is an organic material, it is thermally labile and decomposes in air to CO_2_ and water at high temperatures (>250 °C), which can cause particle aggregation. To prevent aggregation and retain cubic morphology, the nanocubes were separated by embedding them in a silica aerogel support matrix. This should, in principle, allow for continued activity at higher temperatures.

Here, we demonstrate that this approach of embedding ceria nanocubes in an aerogel support matrix can increase the temperature at which the active material can undergo reversible redox. Notably, re-reduction of cerium in the aerogel nanocomposite was achieved at 275 °C after oxidation without the need for additional thermal energy, which was not possible with the unsupported ceria nanocubes. Oxidation is more energetically favorable in comparison to reduction; therefore, reduction can be improved by introducing additional thermal energy. Evidence obtained by transmission electron microscopy that the ceria nanocubes are well separated in the supporting aerogel matrix, as published elsewhere,^10^ thus preventing particle aggregation, also supports the interpretation that a greater amount of the more reactive surfaces is exposed and the surface oxygen can be more readily reduced.

## 5 Conclusions

Ceria nanocubes, La-doped ceria nanocubes, and ceria nanocubes dispersed in a silica aerogel matrix were investigated by using ex situ and in situ Ce L_3_-edge HERFD-XANES in order to observe their redox properties. The much higher resolution of the HERFD-XANES spectra with respect to those obtained by conventional XANES allows for detailed quantification of the Ce^3+^ and Ce^4+^ relative amounts under varying conditions. Static measurements of each material showed doping the nanocubes with lanthanum had similar effects to thermally treating the undoped nanocubes, increasing the oxidation state of the cerium. The in situ experiments demonstrate that embedding the ceria nanocubes into silica aerogels produces enhanced reactivity due to the surfactant being removed while avoiding the growth of the nanocrystals. The improved ceria redox capacity can be understood as being due to the preservation of the cuboidal shape. Consequently, the aerogel nanocomposites maintain thermal stability and show high reactivity due to the accessibility of highly reactive {100} surface facets.

## Supporting Information Available

Choice of Ce(NO_3_)_3_·6H_2_O as Ce^3+^ standard, self-absorption and beam damage effects, linear combination fitting of HERFD-XANES data of CeO_2_ nanocubes, in situ HERFD-XANES of the Ce^4+^ standard, in situ HERFD-XANES of ceria nanocubes, and in situ HERFD-XANES of ceria-aerogel nanocomposites (PDF)
