# Supplementary material for: Using Ex Situ and In Situ HERFD-XANES to Reveal the Superior Oxidation and Reduction Cycling of Ceria Nanocubes Dispersed in Silica Aerogel
Source: J Phys Chem C Nanomater Interfaces. 2023 Sep 21;127(39):19554–62. doi: 10.1021/acs.jpcc.3c03785 (PMC10561250; doi:10.1021/acs.jpcc.3c03785)
Supplement: Supplementary file 1 — jp3c03785_si_001.pdf [file jp3c03785_si_001.pdf]

Supporting Information

Using *Ex-Situ* and *In-Situ* HERFD-XANES to Reveal the Superior Oxidation and Reduction Cycling of Ceria Nanocubes Dispersed in Silica Aerogel

Lucy M. Morgan,[a] Danilo Loche,[a] Anna Corrias,[a] Shusaku Hayama,[b] and Gavin Mountjoy*[c]

[a] School of Chemistry and Forensic Science, Ingram Building, University of Kent, Canterbury CT2 7NH, United Kingdom.
[b] Diamond Light Source, Harwell Science & Innovation Campus, Didcot OX11 0DE, United Kingdom
[c] School of Physics and Astronomy, Ingram Building, University of Kent, Canterbury CT2 7NH, United Kingdom.

* Corresponding author. E-mail address: g.mountjoy@kent.ac.uk (G. Mountjoy)

Choice of $Ce(NO_3)_3{\cdot}6H_2O$ as Ce^{3+} standard

The signature of Ce^{3+} is a very pronounced white line at the Ce L_3-edge which is absent for Ce^{4+}, due to the different electronic characters of the empty states. A detailed comparison of several Ce^{3+} standards (phosphate, carbonate, nitrate, sulfate, and chloride) [S1] shows that the white lines have the same energy position, and have amplitudes that vary by only ±10% about the mean. Furthermore, [S1] states that "Previous studies also agree that the choice of the Ce^{3+} host-phase is not crucial for linear combination fitting analysis" [S1]. The standard used in this study, $Ce(NO_3)_3{\cdot}6H_2O$, has the largest amplitude of the white line, hence the results of the linear combination fitting analysis presented in this work provide a lower bound to the proportion of Ce^{3+}.

It may be noted that $Ce(NO_3)_3{\cdot}6H_2O$ has Ce^{3+} with a coordination number of 11 and Ce-O bond lengths of 2.49-2.88 Å. This compares with Ce^{4+} in CeO_2 having a coordination number of 8 with Ce-O bond lengths of 2.34 Å. The coordination number of Ce^{3+} in reduced CeO_2 is expected to be lower than 8 due to the presence of oxygen vacancies, and in Ce_2O_3 the coordination number of Ce^{3+} is 7. However, the pronounced white line at the Ce L_3-edge is overwhelmingly a density of states effect that originates from Ce^{3+} empty 5d states, and effects due to local atomic environment are subtle and difficult to establish. This is illustrated in reference [S1] where the white line intensity for Ce chloride is the same as for Ce nitrate, despite the Ce^{3+} having very different coordination numbers of 7 and 11 respectively.

[S1] M.Y. Stuckman, C.L. Lopano, and E.J. Granite, Distribution and Speciation of Rare earth Elements in Coal Combustion by-products via Synchrotron Microscopy and Spectroscopy, *Intern. J. Coal Geol.* 2018, **195**, 125–138.

Self-absorption and beam damage effects

For ex-situ measurements the samples were mixed in PVP to achieve a factor of 25x wt% dilution, or 100x mol% dilution. This meant that even for concentrated samples such CeO_2, the effective absorption due to Ce was only a few percent of the total absorption, and any self-absorption is expected to be a minor effect. This is confirmed by excellent agreement between our HEXRD measurements of $Ce(NO_3)_3 \cdot 6H_2O$ and CeO_2 and those reported in [S2]. For in-situ measurements the samples were highly dispersed on quartz wool, resulting in effectively very low concentration of Ce, and self-absorption is expected to be negligible.

The discussion of radiation damage in [S3] states that "The use of low flux (~10^{12} ph/s) minimizes flux dependent (rather than dose-dependent) radiation-induced side effects", and the I20 beamline is expected to deliver a much lower level of flux than this at the Ce L_3-edge, hence limiting beam damage. Other ways to avoid beam damage during Ce L_3-edge measurements are discussed in [S4] and include to cool the sample with liquid nitrogen, use a protective N_2 atmosphere, and an unfocused x-ray beam. However, these precautions are not possible for a study of redox reactions in-situ which requires using elevated temperatures and H_2/O_2 atmospheres, with a capillary, and hence a focused beam.

[S2] A. Manceau, S. A. L. Paul, A. Simionovici, V. Magnin, M. Balvay, N. Findling, M. Rovezzi, S. Muller, D. Garbe-Schönberg, and A. Koschinsky, Fossil Bioapatites with Extremely High Concentrations of Rare Earth Elements and Yttrium from Deep-Sea Pelagic Sediments, *ACS Earth Space Chem.* 2022, **6**, 2093−2103.

[S3] L. Bertrand, S. Bernard, F. Marone, M. Thoury, I. Reiche, A. Gourrier, P. Sciau, U. Bergmann, Emerging Approaches in Synchrotron Studies of Materials from Cultural and Natural History Collections, *Top. Curr. Chem.*, 2016, **374**,7.

[S4] G. Ratié, D. Vantelon, M. Pédrot, A. Beauvois, K. Chaouchi, C. Fossé, M. Davranche, Cerium anomalies in riverbanks: Highlight into the Role of Ferric Deposits, *Sci. Total Environ.* 2020, **713**, 136544.

Linear combination fitting of HERFD-XANES data of CeO_2 nanocubes

The HERFD-XANES data of CeO_2 nanocubes were fitted using a linear combination of Ce^{3+} ($Ce(NO_3)_3·6H_2O$) and Ce^{4+} (CeO_2) standards. Figure S1 shows the experimental data, the fitting, the relative contributions of the two standards, and the residual. It can be noticed that the residual is very low in the region where the spectrum is dominated by the Ce^{4+} standard, and it is more pronounced in the region dominated by the Ce^{3+} standard. This is not surprising because the Ce^{3+} environment in the standard is not exactly the same as in the sample. However, considering the unavailability of a more suitable Ce^{3+} standard, the fitting quality is high enough to provide a reliable quantification of the Ce^{3+} and Ce^{4+} relative amounts.

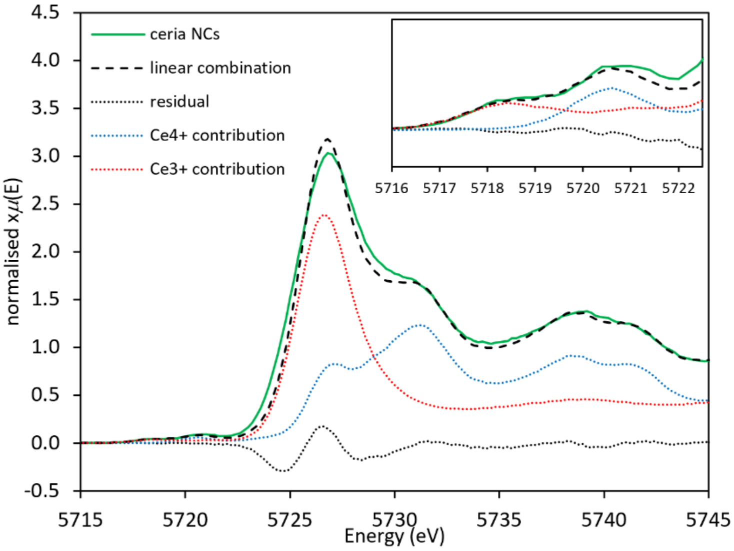

Figure S1. HERFD-XANES Ce L_3-edge spectra of the ceria nanocubes (green) and their fitting as a linear combination (dashed black) of Ce^{4+} (CeO_2) (dotted blue) and Ce^{3+} ($Ce(NO_3)_3·6H_2O$) (dotted red) standards. Also shown is the residual (dotted black). The inset presents a magnified pre-edge.

In-situ HERFD-XANES of the Ce^{4+} standard

In-situ HERFD-XANES Ce L_3-edge measurements of the Ce^{4+} standard (CeO_2) were carried out, Figure S2. Measurements were taken at room temperature under N_2 (green), at 400 °C under H_2 (purple) then O_2 (orange), and finally back to room temperature under N_2 (pink). The temperatures, conditions, and relative ratio of Ce^{3+}/Ce^{4+} are presented in Table S1.

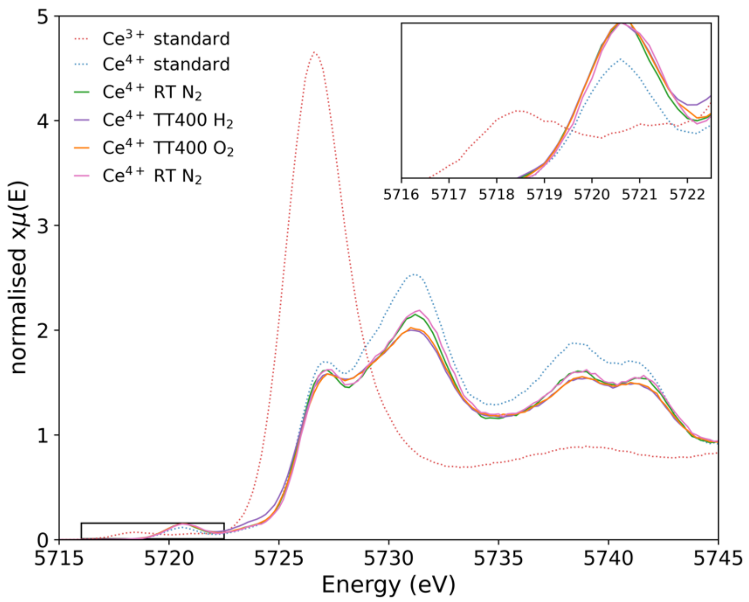

Figure S2. HERFD-XANES Ce L_3-edge spectra of the Ce^{4+} standard (CeO_2) under N_2 (green), at 400 °C under H_2 (purple) then O_2 (orange), and finally back to room temperature under N_2 (pink). The static measurements of Ce^{3+} (dotted red) and Ce^{4+} (dotted blue) standards are also shown. The inset presents a magnified pre-edge.

Table S1. The estimated relative concentrations of Ce^{3+} and Ce^{4+} within the Ce^{4+} standard (CeO_2) during *in-situ* measurements taken at room temperature and 400 °C under reduction/oxidation conditions.

		Concentration (%)	
Ce^{4+} standard	Gas flow	Ce^{3+}	Ce^{4+}
RT	N_2	7	93
400 °C	H_2	9	91
400 °C	O_2	8	92
RT	N_2	5	95

In-situ HERFD-XANES of ceria nanocubes

In-situ HERFD-XANES Ce L_3-edge measurements of ceria nanocubes was carried out. Figure S3 gives the measurements taken at room temperature under N_2 (green), followed by 150 °C under H_2 (purple) then O_2 (orange), then up to 275 °C under H_2 (pink). Figure S4 gives the measurements of 275 °C under H_2 (green) then O_2 (purple), and finally to 400 °C under H_2 (orange) then O_2 (pink), before reducing the temperature back to room temperature under N_2 (grey). The gradual oxidation of the ceria nanocubes over time at 275 °C is given in Figure S5.

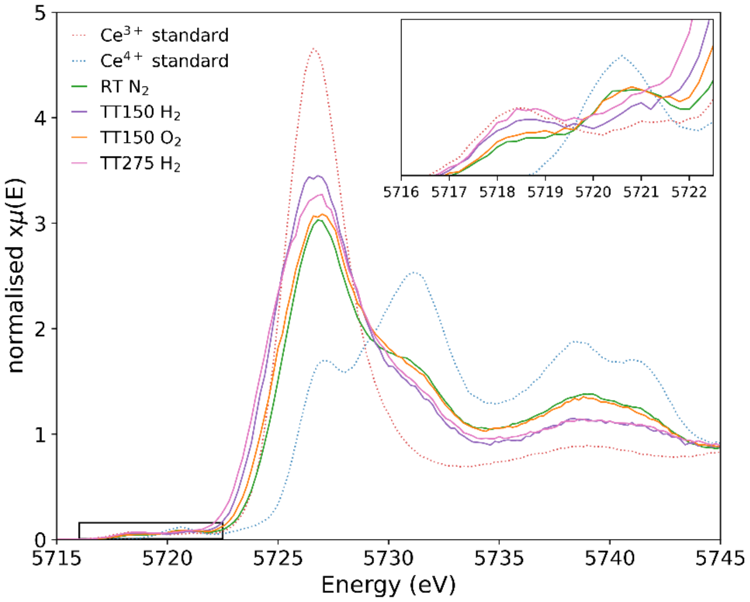

Figure S3. HERFD-XANES Ce L_3-edge spectra of the ceria nanocubes under *in-situ* conditions at room temperature (green), followed by reduction at 150 °C (purple), then oxidation at 150 °C (orange), then reduction at 275 °C (pink). The static measurements of Ce^{3+} (dotted red) and Ce^{4+} (dotted blue) standards are also shown. The inset presents a magnified pre-edge.

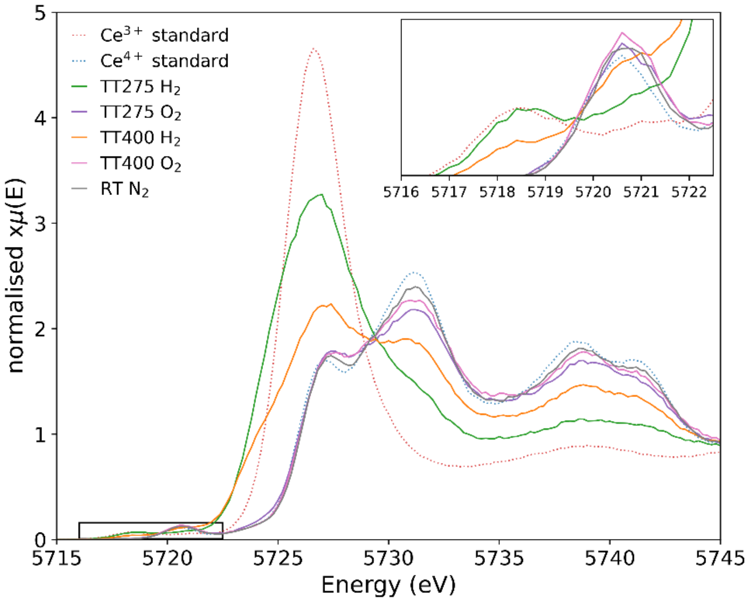

Figure S4. HERFD-XANES Ce L_3-edge spectra of the ceria nanocubes under *in-situ* reduction at 275 °C (green), followed by oxidation at 275 °C (purple), then reduction at 400 °C (orange), oxidation at 400 °C (pink), and finally back at room temperature (grey). The static measurements of Ce^{3+} (dotted red) and Ce^{4+} (dotted blue) standards are also shown. The inset presents a magnified pre-edge.

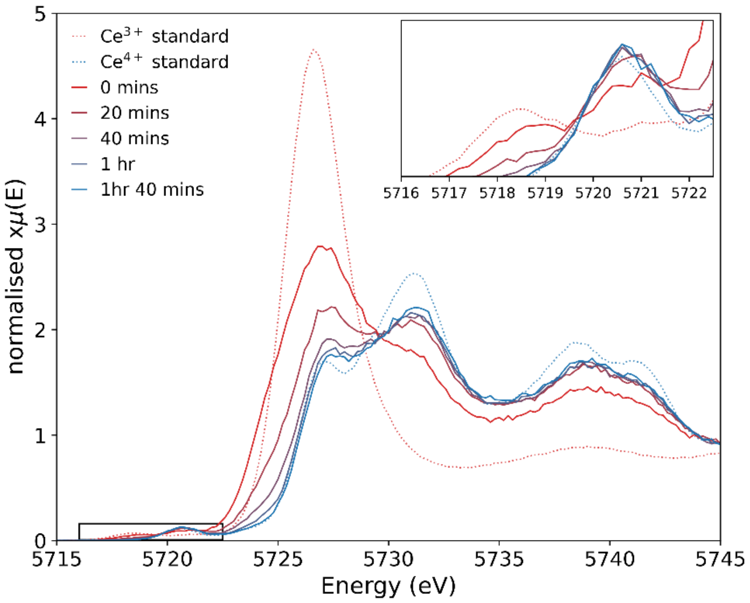

Figure S5. HERFD-XANES Ce L_3-edge spectra of the ceria nanocubes progressively oxidised *in-situ* at 275 °C (from red to blue). The static measurements of Ce^{3+} (dotted red) and Ce^{4+} (dotted blue) standards are also shown. The inset presents a magnified pre-edge.

In-situ HERFD-XANES of ceria-aerogel nanocomposites

In-situ HERFD-XANES Ce L_3-edge measurements of ceria-aerogel nanocomposites were carried out. The gradual oxidation of the ceria at 275 °C is given in Figure S6.

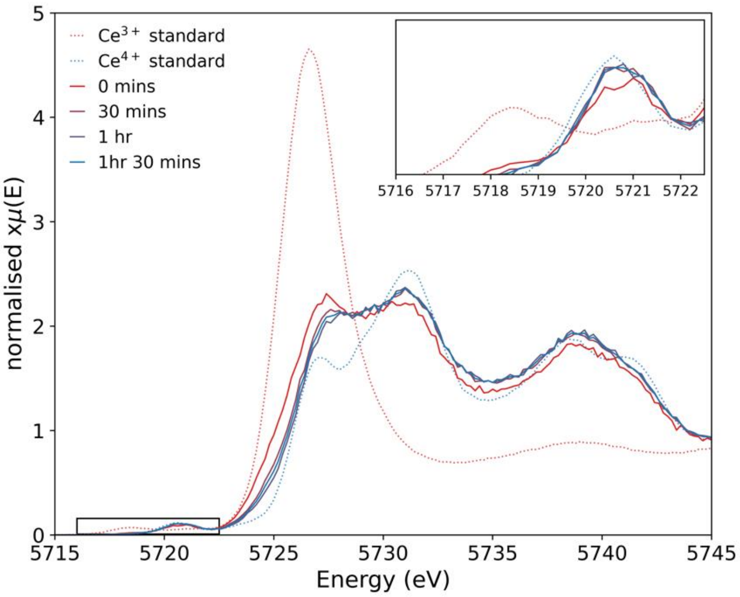

Figure S6. HERFD-XANES Ce L_3-edge spectra of the ceria-aerogel nanocomposite progressively oxidised *in-situ* at 275 °C (from red to blue). The static measurements of Ce^{3+} (dotted red) and Ce^{4+} (dotted blue) standards are also shown. The inset presents a magnified pre-edge.
